# Practical realization of a sub-λ/2 acoustic jet

**DOI:** 10.1038/s41598-019-41335-6

**Published:** 2019-03-26

**Authors:** Daniel Veira Canle, Tuukka Kekkonen, Joni Mäkinen, Tuomas Puranen, Heikki J. Nieminen, Antti Kuronen, Sami Franssila, Tapio Kotiaho, Ari Salmi, Edward Hæggström

**Affiliations:** 1 Department of Physics, Division of Materials Physics, Faculty of Science, P.O.B. 64, FIN-00014, University of Helsinki, Helsinki, Finland; 2 Department of Chemistry and Materials Science, Aalto University, Espoo, Finland; 3 Department of Chemistry, Faculty of Science, P.O.B. 55, FIN-00014, University of Helsinki, Helsinki, Finland; 4 Drug Research Program, Division of Pharmaceutical Chemistry and Technology, Faculty of Pharmacy, P.O.B. 56, FIN-00014, University of Helsinki, Helsinki, Finland; 5 Present Address: Department of Neuroscience and Biomedical Engineering, School of Science, Aalto University, P.O.B. 12200, FIN-00076, Espoo, Finland

## Abstract

Studies in optics and acoustics have employed metamaterial lenses to achieve sub-wavelength localization, *e.g*. a recently introduced concept called ‘acoustojet’ which in simulations localizes acoustic energy to a spot smaller than λ/2. However previous experimental results on the acoustojet have barely reached λ/2-wide localization. Here we show, by simulations and experiments, that a sub-λ/2 wide localization can be achieved by translating the concept of a photonic jet into the acoustic realm. We performed nano- to macroscale molecular dynamics (MD) and finite element method (FEM) simulations as well as macroscale experiments. We demonstrated that by choosing a suitable size cylindrical lens, and by selecting the speed-of-sound ratio between the lens material(s) and the surrounding medium, an acoustic jet (‘acoustic sheet’) is formed with a full width at half maximum (FWHM) less than λ/2. The results show, that the acoustojet approach can be experimentally realized with easy-to-manufacture acoustic lenses at the macroscale. MD simulations demonstrate that the concept can be extended to coherent phonons at nanoscale. Finally, our FEM simulations identify some micrometer size structures that could be realized in practice. Our results may contribute to starting a new era of super resolution acoustic imaging: We foresee that jet generating constructs can be readily manufactured, since suitable material combinations can be found from nanoscale to macroscale. Tight focusing of mechanical energy is highly desirable in e.g. electronics, materials science, medicine, biosciences, and energy harvesting.

## Introduction

A fundamental barrier in wave physics for focusing energy is the diffraction limit. Both in optics and acoustics the Rayleigh criterion^1^ sets the lateral resolution limit in the far-field to λ/2. Several attempts to reach focal spots narrower than λ/2 were listed by Maznev^2^ *et al*. and include the modification of the numerical aperture of the system, the use of metamaterial lenses, and focusing structures such as antennas. The use of metamaterial lenses is common practice both in optics and acoustics^3–5^. Theoretically^6^, one can focus acoustic energy by means of a metamaterial lens that focus acoustic energy into a jet. Experimentally, it has been shown that it is possible to reach λ/2-wide focal spots with such a lens^7–9^. This study shows that by cleverly selecting the lens and surrounding medium materials, as well as the wave frequency and the lens size, it is possible to localize acoustic energy to a spot smaller than λ/2.

The idea for this study emerged from our research on photonic jets^10^. The seminal paper by Chen *et al*.^11^ shows, that one can achieve super resolution with light by cleverly selecting the refractive indices in the lens and in the surrounding medium. They obtained super resolution when two conditions were met: The refractive index ratio was approximately 1.7 and the lens was 20–30 wavelengths in diameter. This prediction was supported by ray tracing studies in the optics realm of monochromatic plane waves impinging on a dielectric cylinder as shown by Adler *et al*.^12^. This concept was translated to the acoustic domain, first theoretically^6^ and then in practice by Lopes *et al*. and Minin *et al*.^7–9^, who never achieved sub-λ/2 localization experimentally. Here, we show that it can be achieved. In photonic jet research, the surrounding medium should have a smaller refractive index than the lens, which indicates that the speed of light in the lens should be lower than in the medium:

1 $${n}_{lens} \sim 1.7{n}_{medium}\to \frac{c}{{v}_{lens}} \sim \frac{1.7c}{{v}_{medium}}\to {v}_{lens} \sim \frac{1}{1.7}{v}_{medium}$$

where *n* is the refractive index, *c* is the speed of light in vacuum, and *v* is the speed of light in the medium surrounding the lens.

Previous super resolution approaches in the literature include using metamaterial lenses. A metamaterial lens features a periodically ordered arrangement of unit cells spaced at subwavelength distance from each other. As a result, the lens can feature effective negative density and compressibility^13^. This remarkable feature allows the lenses to transform evanescent waves into propagating waves. Since evanescent waves feature much shorter wavelength than the travelling wave, the contrast of the imaging technique is improved^14,15^. However, metamaterial lenses usually rely on narrowband resonances^4,13^. To circumvent this limitation, Kaina *et al*. suggested to use a broad band excitation in combination with time reversal to generate subwavelength focal spots^14^. Ma *et al*. used a combination of a metamaterial structure that acted as an acoustic sink in combination with time reversal to focus acoustic energy into a subwavelength region inside the metamaterial structure^16^. Our approach is to generate a line focus (‘acoustic sheet’) instead of a jet generated by a sphere, since cylindrical structures can be readily manufactured across many length scales.

**Figure 1:**
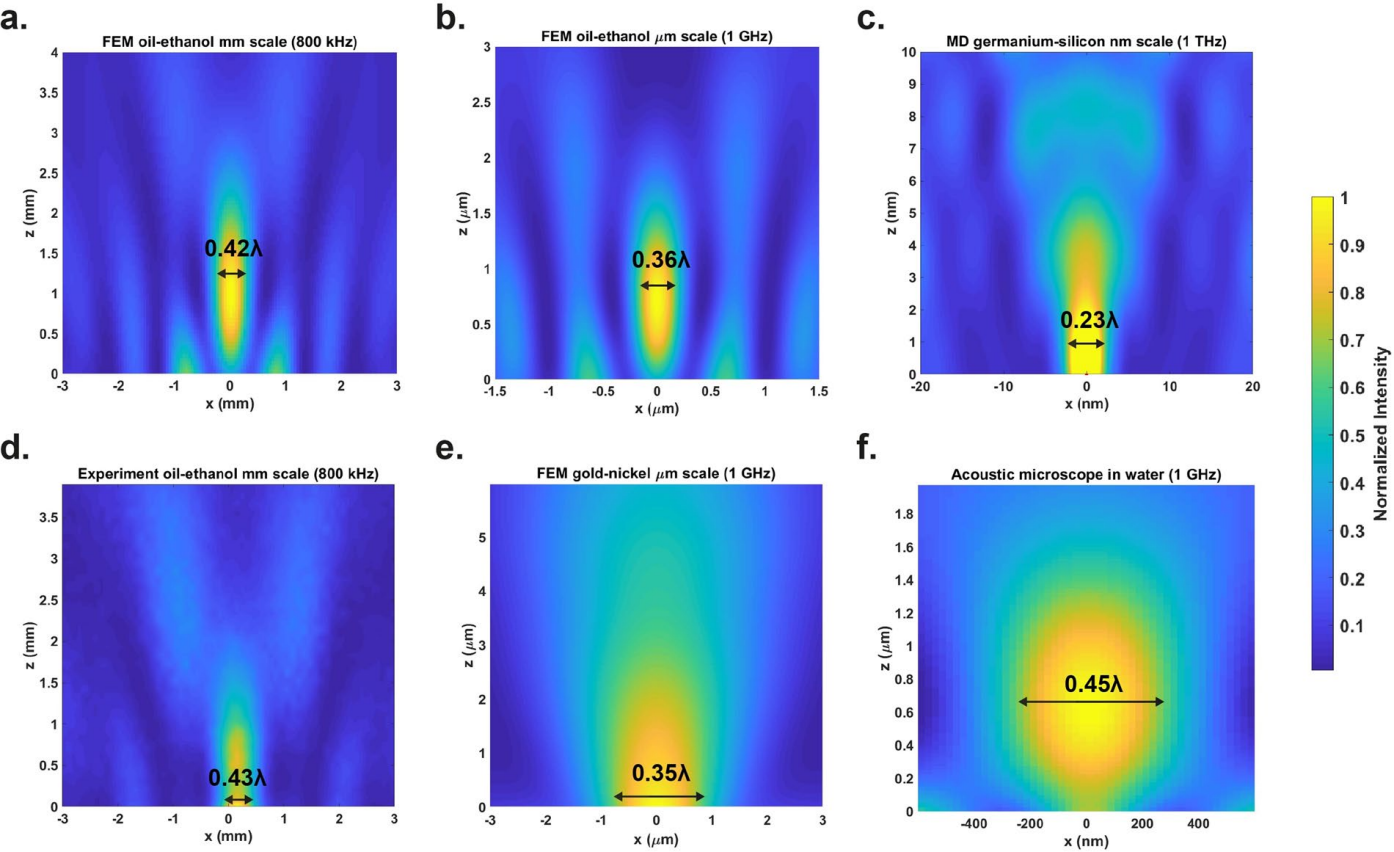
Sub-λ/2 localization. Acoustic fields obtained in simulations (**a–c**, **e,f**) and experimentally (**d**). The acoustic jet FWHM is marked in each figure as multiples of the excitation wavelength. We performed simulations across many length scales: (**a**) FEM simulation of an acoustic jet at millimeter scale, excitation frequency 800 kHz. This prediction was experimentally validated, as seen in (**d**) featuring an acoustic jet experimentally measured in ethanol at 800 kHz. We also simulated the jet structure at micro- and nanoscale: (**b**) FEM simulation of a liquid-liquid core shell acoustic jet in ethanol at 1 GHz; (**e**) FEM simulation for a solid-solid lens structure at 1 GHz and at nanoscale: (**c**) MD simulations of an acoustic jet created with incoming coherent phonons at 1 THz. (**f**) FEM simulation of an acoustic jet structure applicable for acoustic microscopy.

First, we ran simulations, both MD and FEM, over six orders of magnitude in length (mm to nm). Our FEM predictions indicated that sub-λ/2 localization can be achieved across the acoustic frequency spectrum by scaling the size of the structure. We built two kinds of simulation models (lens material – surrounding media): a liquid-liquid core-shell lens whose dimensions are in the cm-µm scale (Figs S1, S2a,b), and a solid-solid lens whose dimensions are in the µm-nm scale (Fig. S2c). In addition, we simulated a µm scale construct applicable for acoustic microscopy (solid-water, Fig. S2d). These results are shown in Fig. 1: the FWHM of the acoustic pressure squared (proportional to intensity) was 0.42 λ at 800 kHz (Fig. 1a), 0.36 λ at 1 GHz for a liquid-liquid core-shell lens (Fig. 1b), 0.45 λ at 1 GHz for the construct applicable for acoustic microscopy (Fig. 1f), and 0.35 λ for the solid-solid lens at 1 GHz (Fig. 1e). Finally, we built a MD model to demonstrate that the technique is feasible also for coherent phononic use: we excited 1 THz coherent phononic plane waves which impinged on the structure (Figs S4 and S5) and generated a 1.9 nm FWHM (0.23 λ) phononic sheet, Fig. 1c.

**Figure 2:**
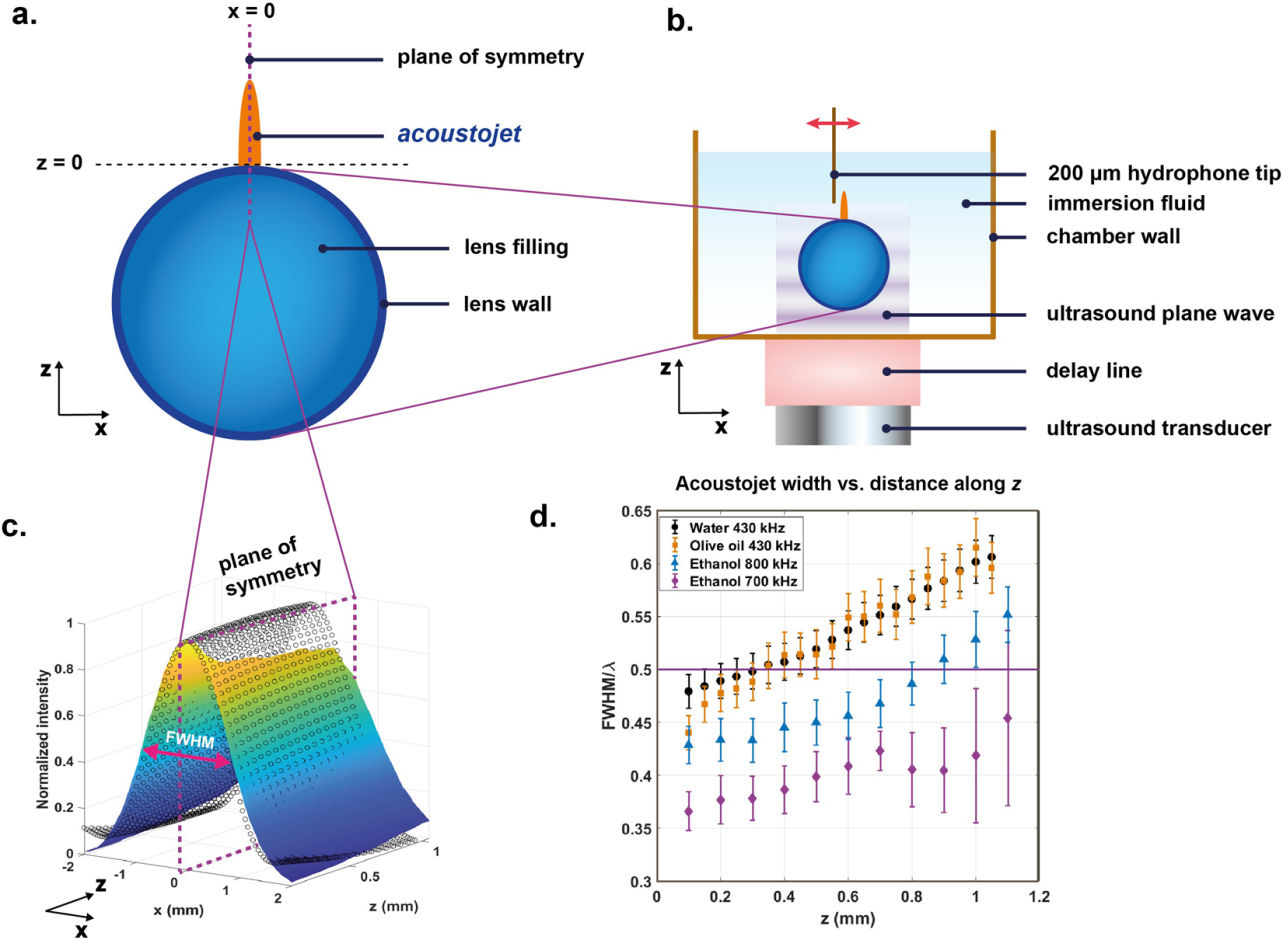
Experimental realization of the acoustic jet. (**a**) The core-shell lens structure consisted of a polyethylene sample tube (shell) and perfluorinated oil (core). The generated acoustic jet is symmetric in the xz plane. (**b**) Experimental setup: plane waves emerge from a delay line and travel through the lens that produces an acoustic jet that is probed by a 200 µm diameter needle hydrophone. The spatial resolution of the experimental results is limited by the probe diameter. (**c**) The experimentally measured acoustic jet (surface plot) validates the simulated prediction (open circles) in water at 100 µm distance from the lens surface. (**d**) FWHMs smaller than λ/2 (purple line) are seen for olive oil and ethanol as surrounding media. The error bars indicate confidence limits of one standard deviation.

**Figure 3:**
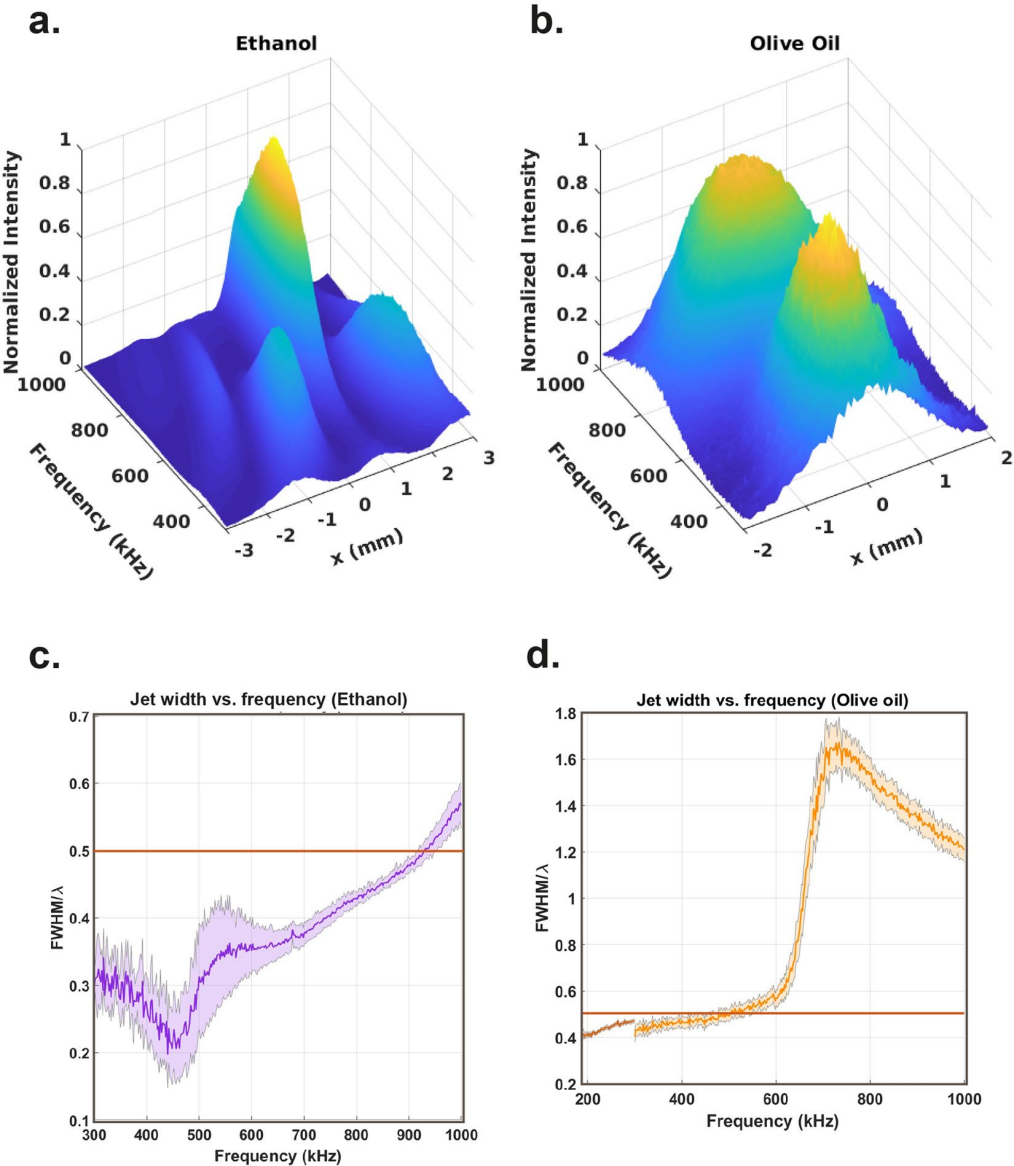
Frequency dependence of the experimentally generated acoustic jet. Jet shape in the measured acoustic field as a function of frequency for (**a**) perfluorinated oil-ethanol and (**b**) perfluorinated oil–olive oil constructs. The jet width is narrower than λ/2 (orange lines) for a wide range of frequencies, (**c,d**). The shaded error bars represent confidence limits of one standard deviation. Two transducers of different bandwidths (orange and brown) were used in the frequency sweep of (**d**).

We validated our simulation predictions (Fig. 1a) by performing measurements on mm-cm scale (Fig. 1d). Our experimental setup (Fig. 2a,b) featured a core-shell lens structure with a thin polymer cylinder filled with perfluorinated oil that has a relatively low speed of sound, (Table S1). Since the photonic jet theory and the experiments employ incoming plane waves, we attached a delay line between the transducer and the experimental chamber (Fig. 2b) to ensure that waves propagating through the immersion medium satisfy the plane wave approximation. The surrounding media into which the jet is generated was varied: we selected three candidates, out of which two were biocompatible (water and olive oil) and one was not (ethanol). We tested several speed of sound ratios (which are analogous to the speed of light ratio, Eq. 1) between the lens material and the immersion fluid both in FEM simulations (Fig. S3) and experimentally (Fig. 2d). The narrowest FWHM was expected for the ethanol-perfluorinated oil combination (speed of sound ratio = 1.6), followed by the olive oil – perfluorinated oil combination (speed of sound ratio = 2.0), and finally the water-perfluorinated oil (speed of sound ratio = 2.1). The experimental results validate the predictions (Figs 1d and 2c,d). We examined the FWHM as a function of the wavelength of the incoming wave by performing frequency scans for the ethanol and olive oil cases, Fig. 3. These scans demonstrate that the FWHM of the generated jet is well below λ/2 for a wide range of frequencies. Consequently, the applied frequency can be slightly altered without major effect on the resolution.

The experimental results obtained on macroscale should translate to smaller scales: when the lens size is reduced (and the frequency is increased) while maintaining the speed of sound ratio, our simulations predict no hard-lower limit for how narrow the acoustic jet can be. Such scaling down of the lens size to µm-, and even nm scale should be feasible by micro- and nanofabrication techniques. However, there are phenomena present at microscale (such as microscopic fluid flow and thermoviscous effects) that the FEM simulations in this study did not take into account.

In principle, the MD simulated (Fig. 1e) construct could be experimentally tested with a 325 GHz phononic ‘laser’^17^ structure, as long as the lens size is scaled up accordingly (diameter of the lens ~25 λ, for 325 GHz this is approximately 400 nm). For liquid core-shell lenses, such as those in Fig. 2, biocompatible immersion media exist. Hence the technique can be applied to image biological objects. If biocompatibility is not needed, a greater variety of combinations can be utilized, *e.g*. gold-nickel, ethanol-perfluorinated oil, and silicon-germanium. We also hypothesize that our simulation and experimental results may generalize into spheres which would allow moving from 2D to 3D.

Since acoustic waves travel slower than light, the width of the energy focus that one can theoretically obtain is narrow, even sub-nanometer (Fig. 1e). Acoustic waves carry the intrinsic advantage compared to light that they can directly measure mechanical properties and can actuate materials without causing photo-bleaching or ionization. The type of tight focusing of mechanical energy demonstrated here is highly desirable and could open up new fields such as high-resolution characterization and/or modification of scientifically and industrially relevant surfaces with focused coherent phonons.

## Methods

### Simulations

We performed FEM simulations of the acoustic field using COMSOL Multiphysics® (version 5.2). The simulations were done at millimeter and micrometer scale. The simulation geometry representing the experimental case (mm scale) and that of the micrometer scale, consisted of the immersion fluid (water, olive oil or ethanol) and a polyethylene shell filled with perfluorinated oil (Figs S1 and S2a). The density and speed of sound of these media were determined experimentally (Table S2). The micrometer scale simulations for the metal structures consisted of a metal cylinder embedded in another metal (Fig. S2c,d). The simulations were done in the frequency domain and a 2D geometry was used (Figs S1 and S2). COMSOL’s implementation of the acoustic-structure interaction was used in cases that featured fluids. A pressure boundary condition at the bottom edge of the geometry was employed to create the acoustic waves (Supplementary Video 1). To minimize acoustic reflections, we replaced the other boundaries of the geometries with perfectly matched layers or radiating boundaries. The mesh size of the geometry was refined to resolve the expected resolution of <λ/2 (Fig. S1).

MD simulations were performed using LAMMPS^18^. The simulated system featured a 114 × 114 × 2.2 nm^3^ slab of silicon with a germanium cylinder (diameter of 28 nm) embedded in the center (Fig. S4). The materials were chosen such that the ratio of their speed of sound corresponded to that used in the experiment. The interaction model for germanium was modified so that its lattice constant was the same as for silicon. This was done to minimize the effect of interfacial stresses. The system was relaxed by minimizing its potential energy. The potential model used in the simulations describes well the structural and elastic properties of Si and Ge and their alloys according to Balamane and Laradji^19,20^. All acoustic simulations were performed using this system and zero Kelvin temperature. The acoustic excitation was a plane wave in the form of a Gaussian wave packet with an amplitude of 5 × 10^−4^ nm, a frequency of 0.6–1.6 THz, and a Gaussian FWHM of 7 ps (Supplementary Video 2).

### Experiments

Our experimental setup (Fig. 2) employed a cylindrical metamaterial core-shell lens glued into a 3D-printed (polylactic acid) chamber (Fig. S9) with epoxy (Araldite® extra strong). The shell was a polyethylene tube (outer diameter 30 mm, 1 mm wall thickness, Electron Microscopy Sciences, model 64240–05), and the core was a liquid, whose speed of sound was less than that of the immersion medium. The selection of the lens material and the surrounding medium was based on their practical usability (toxicity, reactivity, biocompatibility) and their speed of sound ratio. Two of the liquid media surrounding the lens were biocompatible (water and olive oil), one was not (ethanol 99.6%) whereas the liquid core inside the core-shell lens was perfluorinated oil (Fomblin YL-VAC 25–6), which features a low speed of sound (Table S2).

A 10 cm long acrylic block acted as an acoustic delay line to allow doing the measurements in the far field of the transducer where the acoustic field features plane waves. These ultrasonic waves (430 kHz, 5 cycles, 164 V_PP_) were generated with a Karl Deutsch S24 HB 0.3–1.3 MHz transducer coupled with ultrasound transmission gel (Aquasonic 100) to the delay line. We used burst transmission to avoid contribution from wall/surface echoes. To pick up the propagating waves, we used a hydrophone (Precision Acoustics SN 2151, 200 μm probe) attached to a translation stage (Isel Inc.) for scanning. We performed our experiments in the xz plane (Fig. 2) due to the cylindrical symmetry of the acoustic sheet. First, we lowered the hydrophone so that it slightly touched the surface of the cylinder and then we retracted it 100 μm. Next, we filled the container with test medium and excited the acoustic waves. The hydrophone recorded the signals while moving in steps of 100 μm in x and 50 μm in z direction. We squared the received pressure signals and integrated over two cycles to produce intensity maps (Figs 1d, 3 and S12). Figure S12 shows that we did not generate second harmonics into the main beam.

## Supplementary information

Supplementary material
