# Supplementary material for: Practical realization of a sub-λ/2 acoustic jet
Source: Sci Rep. 2019 Mar 26;9:5189. doi: 10.1038/s41598-019-41335-6 (PMC6435727; doi:10.1038/s41598-019-41335-6)
Supplement: Supplementary file 1 — Supplementary material [file 41598_2019_41335_MOESM1_ESM.pdf]

SUPPLEMENTARY MATERIAL

Practical realization of a sub-λ/2 acoustic jet

Daniel Veira Canle[1], Tuukka Kekkonen[1], Joni Mäkinen[1], Tuomas Puranen[1], Heikki J Nieminen[1,**)], Antti Kuronen[1], Sami Franssila[2], Tapio Kotiaho[3,4], Ari Salmi[1,*)] and Edward Hæggström[1]

[1] Department of Physics, Division of Materials Physics, Faculty of science, P.O.B. 64, FIN-00014 University of Helsinki, Finland

[2] Department of Chemistry and Materials Science, Aalto University, Espoo, Finland

[3] Department of Chemistry, Faculty of Science, P.O.B. 55, FIN-00014 University of Helsinki, Finland

[4] Drug Research Program, Division of Pharmaceutical Chemistry and Technology, Faculty of Pharmacy, P.O. B. 56, FIN-00014, University of Helsinki, Finland

[*)] Corresponding author:

Department of Physics

University of Helsinki

P.O.B. 64

00014 Helsinki

E-mail: ari.salmi@helsinki.fi

Telephone: +358 2941 51199

[**)] Current address: Department of Neuroscience and Biomedical Engineering, School of Science, Aalto University, P.O.B. 12200, FIN-00076 Espoo, Finland.

FEM SIMULATIONS

The FEM simulations were modelled in 2D geometry, the actual geometries used in Fig. E1 are shown in Figs. S1 and S2. Two coupled physics interfaces were used in COMSOL Multiphysics® (v 5.2): the pressure acoustics module for liquids and the solid mechanics module for solid parts. The pressure pulse used in the experiments was approximated to be continuous in the simulations to permit using a frequency domain study instead of a transient study. The frequency domain approach was chosen since it is computationally more efficient than a full transient simulation, especially since we needed to run parametric sweeps across frequencies and geometries. The frequency domain simulations give the same result as the steady state solution for a sufficiently long transient simulation. Because our experiments were done with a pulsed acoustic signal in a finite geometric domain, the frequency domain simulation takes into account some echoes from the structure that are absent in the experimental results. The effect of these echoes was small enough that the frequency domain simulations give a sufficiently accurate representation of the experimentally measured intensity.

The basic structure for the models is shown in Fig. S1. Here the geometry is the one used for ethanol in the centimeter scale, same as Fig. S2a. The geometry consist of a solid, liquid filled tube (Fig. S2a-b) or a solid cylinder structure (Fig. S2c-d), a surrounding medium that is either liquid (Fig. S2a-b, top part of S2d) or solid (Fig. S2c, bottom part of S2d) and a Perfectly Matched Layer (PML) layer on the left, right ,and top, except in Fig S2d where a PML is only in the solid domain. The speed of sounds for materials used in simulations with ethanol, water and olive oil (Fig. 1a-b, Fig. E1a-b, Fig. E2) were obtained experimentally (Table S2), generic values [22,23] were used for the polystyrene cylinder (Table S1). The materials used in the micro scale simulations (Fig. 1c-d, Fig. E1c-d) in metals were from COMSOL's material library (Table S1). A pressure boundary condition was used at the bottom edge of the geometry to produce the acoustic wave. In Fig. S2a the excitation area was set for coordinates between -3 cm to 3 cm and for Fig. S2b-d it was set for coordinates -20 µm to 20 µm. To truncate the domain a PML boundary region was used in all simulations except in the top part of Fig. S2d where a radiating boundary condition had to be used instead.

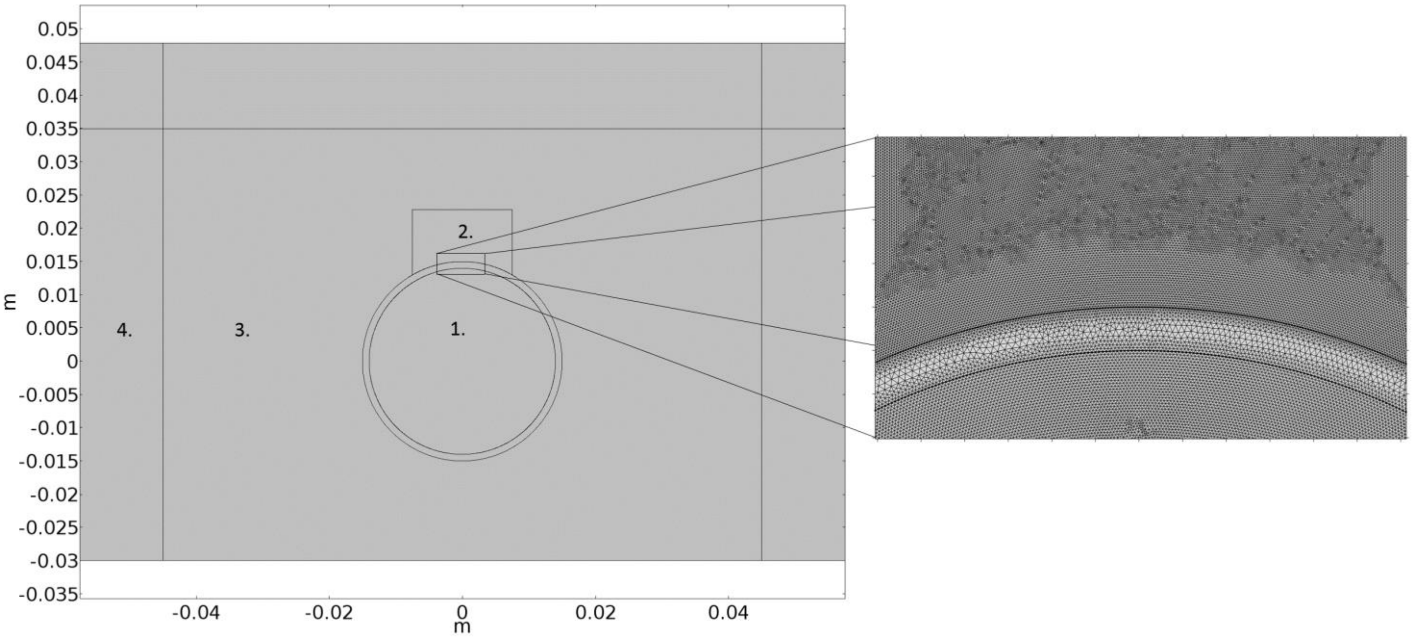

Supplementary Figure 1 - The 2D geometry used in the simulations. Left image: The simulation geometry used in Fig. 1a and in Fig. E1a. Meshing of the domain was done in four parts. The domains where the wave propagates (1-3) were meshed automatically according to the wavelength (λ) in the medium with triangular elements. The edge regions, the PMLs (4), were meshed with a structured mesh that had 12 layers. Mesh size in the regions were: (1) λ/10, (2) λ/20, and (3) λ/6, fulfilling the required minimum resolution of λ/5 to fully resolve a wave with the default 2nd-order Lagrange elements in COMSOL. Right image: Close-up of the mesh used for this geometry.

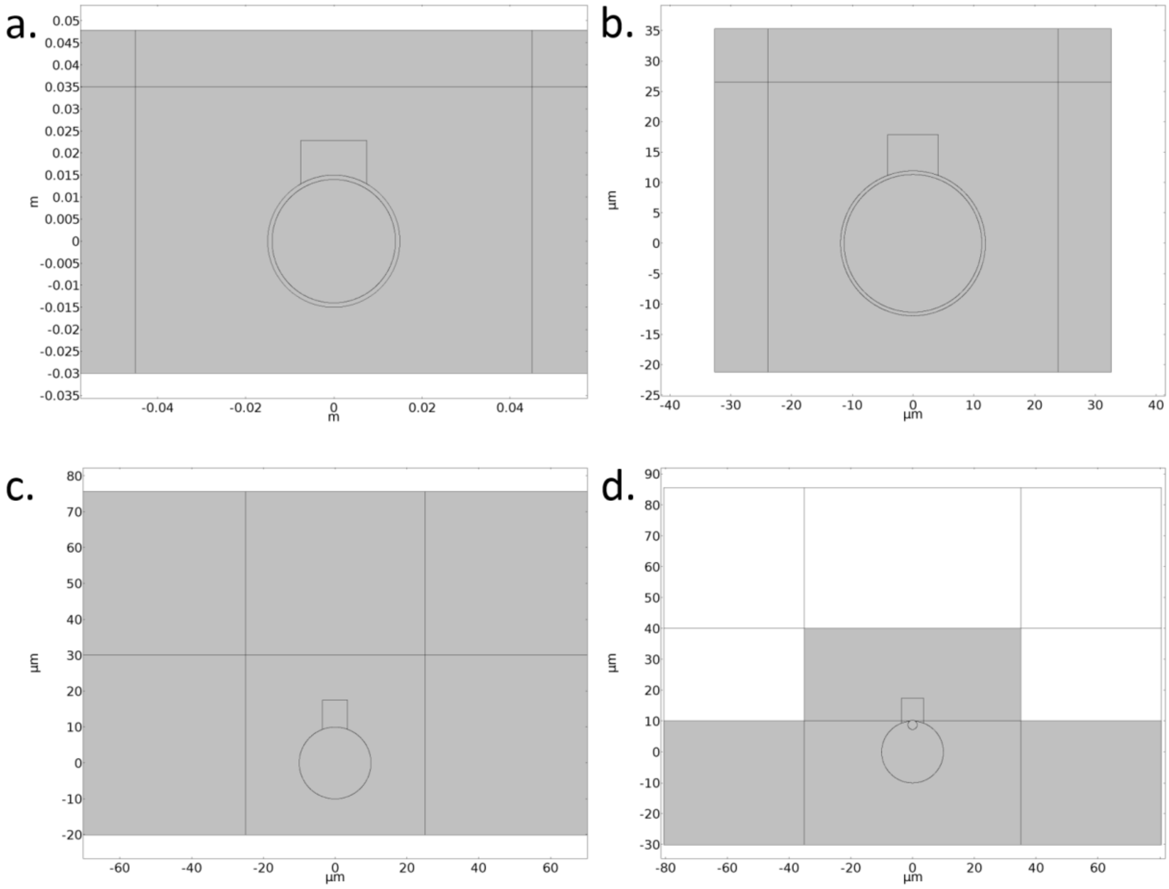

Supplementary Figure 2 - Simulation geometries used to study the acoustic jet at different length scales. a) Ethanol and perfluorinated oil on cm scale (Figs. 1a, E1a). b) Ethanol and perfluorinated oil on µm scale (Figs. 1b, E1b). c) Gold and nickel on µm scale (Figs. 1e, E1c). d) Gold, nickel, lead, and water on µm scale (Figs. 1f, E1d). Note that the box above the cylinders is used for meshing purposes only and that PMLs are used in all geometries except in the water part of picture d) where a radiating boundary is used instead. The cylinder radii are a) 1.5 cm, b) 11.92 µm, c) 10 µm, and d) 10 µm for gold and 1.5 µm for lead.

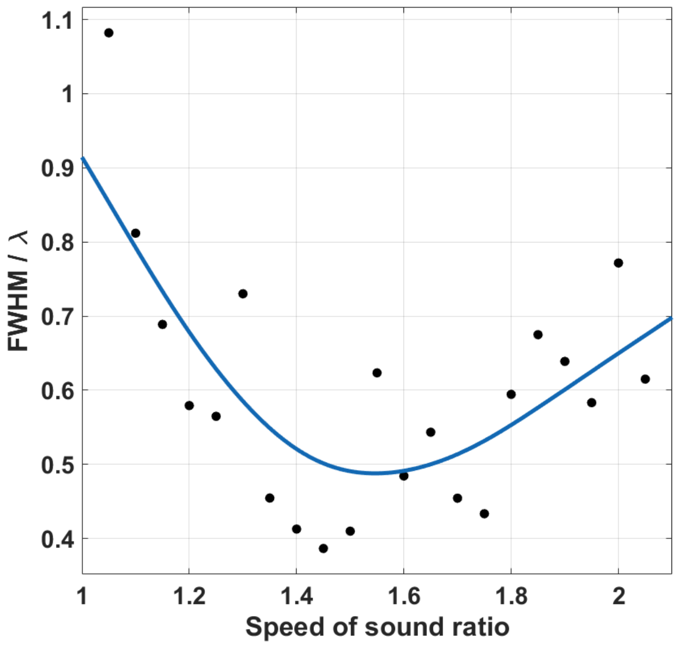

Supplementary Figure 3 – FEM simulation results of the acoustojet width as a function of the speed of sound ratio between the surrounding medium and the lens. There is a local minimum close to 1.6, the region where we performed the experiments with ethanol. The solid blue line is a smoothed spline fit to the simulation data.

Material	Density (kg/m^3)	Young's Modulus (GPa)	Poisson's ratio	Speed of sound (m/s)
Polystyrene	1050	2.9	0.34	2062*
Nickel	8900*	204.7*	0.29*	5466*
Gold	19282*	75.8*	0.44*	3626*
Lead	11340	16.0	0.44	2138*
Water	1000*	-	-	1481*

Supplementary Table 1 - Material properties used in the microscale FEM simulations for metal structures and generic values for the polystyrene used in the ethanol-perfluorinated oil simulations. *Parameter defined as a function of temperature, evaluated at T=293.15 K.

MOLECULAR DYNAMICS (MD) SIMULATIONS

The simulation system was built by creating a diamond slab of size of 210×4×210 (*x*×*y*×z) unit cells of 0.54 nm. The total number of atoms was 1.4×10^6. A cylindrical metamaterial lens 28 nm in diameter featuring germanium atoms was located at the center of the system (Fig. S4). The rest of the system featured silicon atoms. The simulation system was prepared by minimizing the potential energy using the conjugate gradient method. All acoustic simulations were done using this system in which the number of atoms, the volume and the energy remained constant. Periodic boundary conditions were applied along the x and y directions and open boundary conditions along the z direction.

The interaction between the atoms was described by the Stillinger-Weber potential [24]. The parameter σ of the germanium part of the potential was set to the same value as for silicon to minimize the interfacial stresses. Simulations were performed using the LAMMPS [25] MD code. Speed of sound for silicon and germanium used in this work were determined from LAMMPS simulations to be 8328 m/s and 5092 m/s, respectively.

The acoustic wave was generated by moving three bottom layers of atoms according to functional form:

$$\Delta Z = \Delta Z_0 exp(-(t-\mathrm{t}_0)^2/(2\sigma^2))sin(2\pi f t)$$

Where $\Delta Z_0 = 5*10^{-13}\ m$, $t_0 = 4\ ps$, $\sigma = 3\ ps$ and $f = 0.6-1.6\ THz$.

Data analysis and visualization was performed using Ovito [26]. The average hydrostatic pressure squared presented in Fig. 1c in this manuscript was calculated as the time average over 30 ps after the pulse initiation. This time was long enough for the entire pulse to pass through the Ge cylinder but short enough that the wave reflection from the open bottom surface did not affect the results.

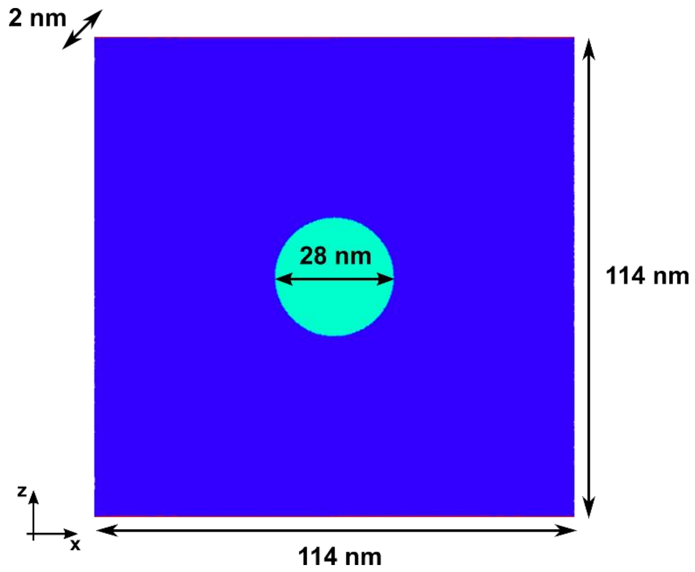

Supplementary Figure 4 - Structure of the molecular dynamics simulation system. Atoms representing the medium (Si) are dark blue whereas atoms constituting the metamaterial lens (Ge) are cyan.

Instantaneous hydrostatic pressure is visualized in Fig. S5. The snap shot is taken 23 ps after wave initiation. A focusing of acoustic energy is seen.

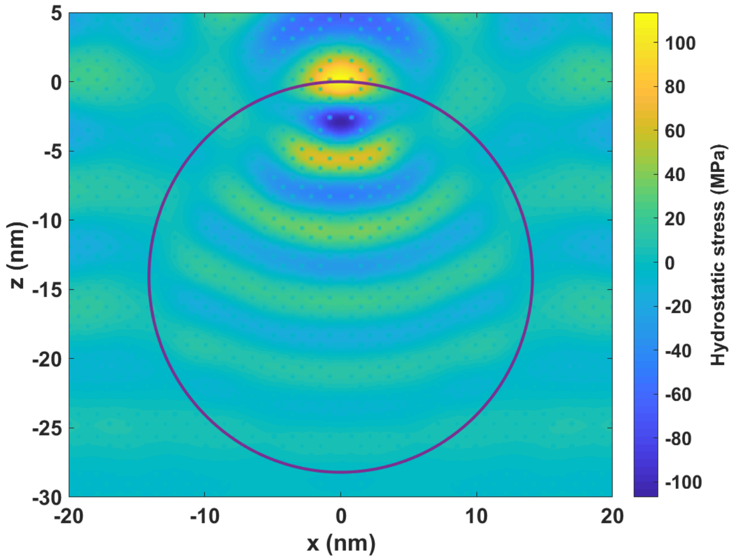

Supplementary Figure 5 - MD simulated stress field. Hydrostatic pressure (not intensity) as obtained from MD simulations (in MPa) at 23 ps after the wave packet initiation. The germanium cylindrical lens is presented as a purple circle. Excitation frequency was 1 THz.

EXPERIMENTS

We measured the speed of sound (Fig. S6) and density (Fig. S7) of the materials at room temperature. A Panametrics 5072PR pulser launched an acoustic pulse through a Karl Deutsch S 24 HB 0,3-1,3 - piezoelectric transducer. The transducer was coupled to a beaker containing the liquid. We varied the distance that the acoustic wave traveled in the liquid by changing the volume of the fluid.

The density measurement was conducted by placing a beaker on top of a precision scale (Precisa 410AM-FR) and varying the volume of fluid in the beaker.

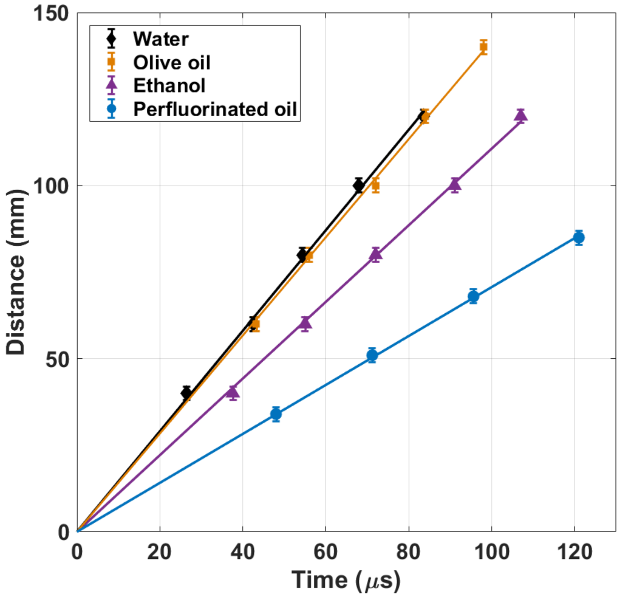

Supplementary Figure 6 - Speed of sound measurement results. The speed of sound is the slope of the linear fit (Table S1). The error bars correspond to twice the thickness of the beaker marks used in the speed of sound measurements. There were no repeats of this experiment.

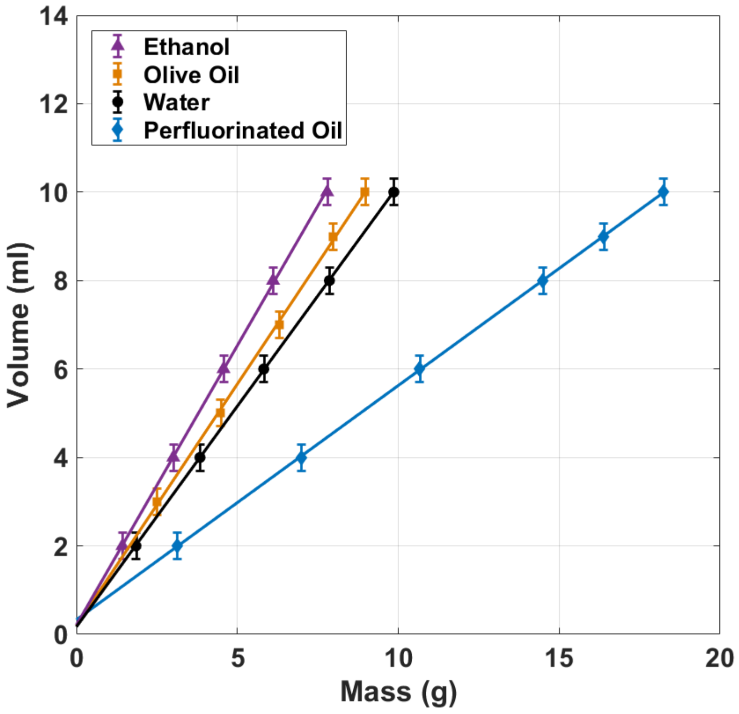

Supplementary Figure 7 - Density measurement results. We measured the mass of different volumes of fluid with a precision scale. The fluid density is the slope of the linear fit (Table S2). The error bars correspond to the volume uncertainty described by the beaker manufacturer. There were no repeats of this experiment.

	Water	Olive oil	Ethanol	Perfluorinated oil
Density (kg/m^3)	1002 ± 9	914 ± 51	791 ± 21	1888 ± 16
Speed of sound (m/s)	1452 ± 33	1418 ± 24	1107 ± 21	707 ± 10

Supplementary Table 2 - Measured density and speed of sound for the studied media at room temperature. The uncertainty limits correspond to two standard deviations.

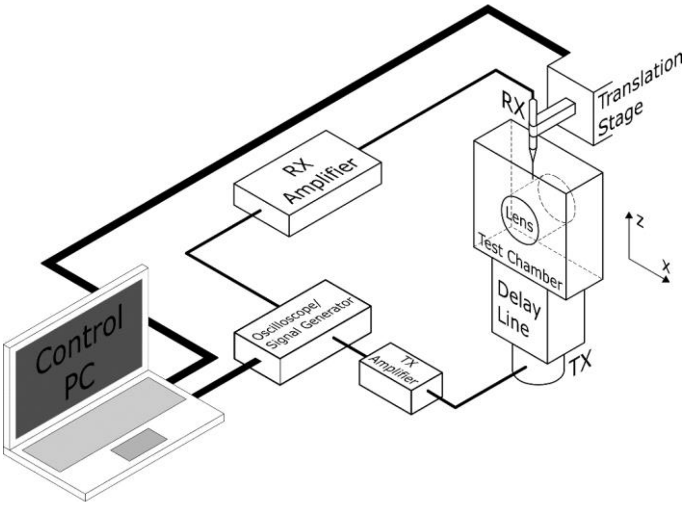

Supplementary Figure 8 - Schematic picture of the experimental setup. We launched acoustic bursts (Digilent: Analog Discovery 2) through a transducer (Karl Deutsch S 24 HB 0,3-1,3) with 164 V amplitude. The acoustic signal was picked up with a hydrophone (Precision Acoustics SN 2151) and was amplified by 60 dB (Panametrics 5660C). The hydrophone was moved between two bursts by a translation stage (Isel: Isert electronic).

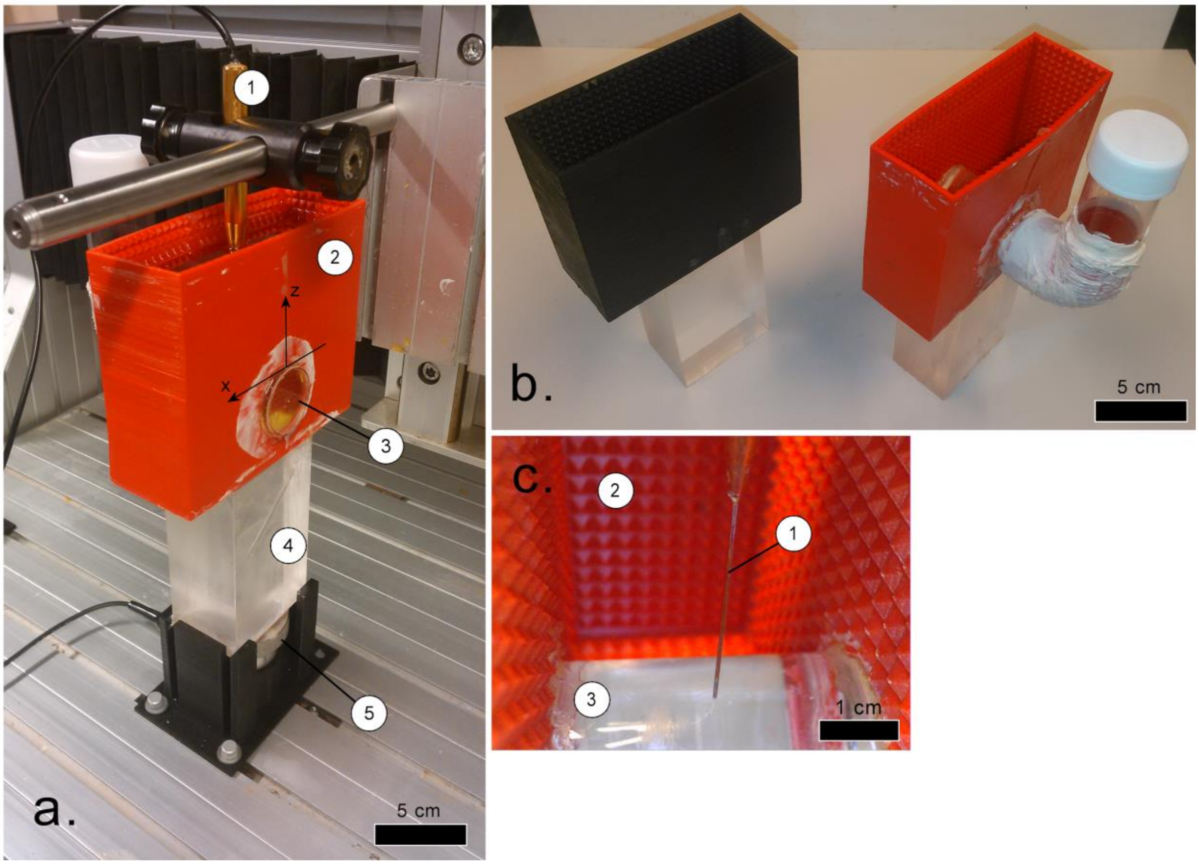

Supplementary Figure 9 - Measurement chamber seen from different view angles. a) Experimental set-up 1) hydrophone RX, 2) test chamber made from polylactide (PLA) and filled with medium of study, 3) polystyrene cylinder filled with perfluorinated oil (metamaterial lens), 4) acrylic delay line, 5) transducer TX. b) Measurement chamber with (right) and without (left) the metamaterial lens. The acrylic block, the test chamber, and the polystyrene cylinder were glued together with Araldite extra strong epoxy. A rectangular hole in the bottom of the test chamber provides direct contact between the acrylic block and the medium of study. c) View into the test chamber. The hydrophone is close to the cylinder without touching it.

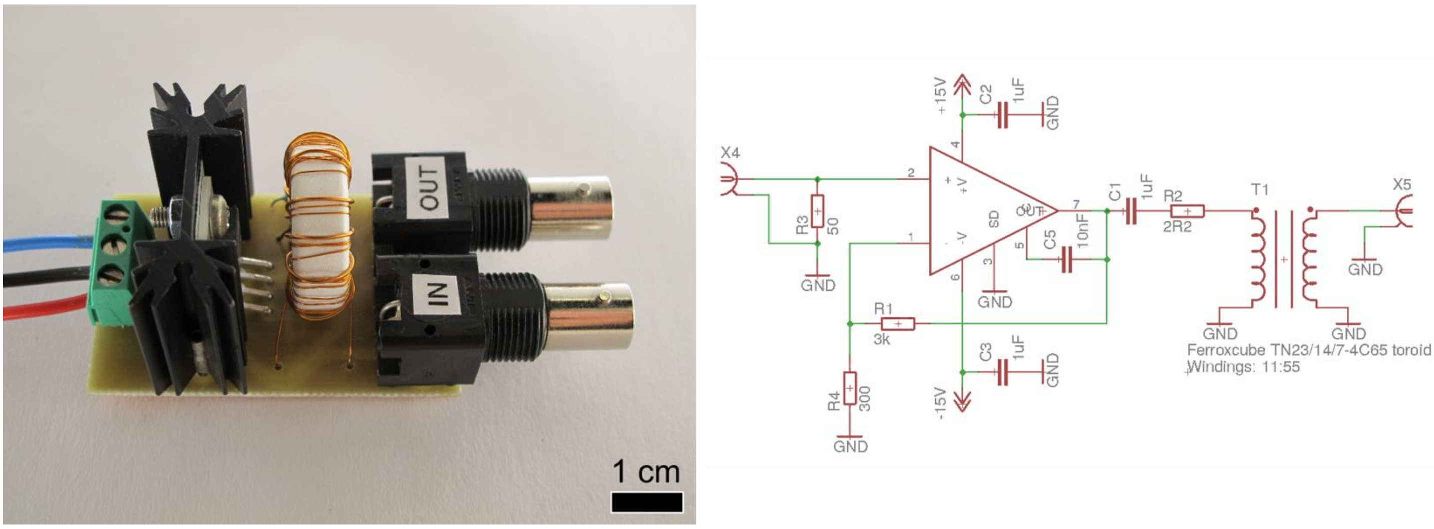

Supplementary Figure 10 - Power amplifier used to drive the TX transducer. The current feedback amplifier (LT120, Linear Technology) and impedance matching transformer are visible in the image.

DATA ANALYSIS

From a single A-line (Fig. S11), we examined a time window of two periods. We did this to minimize the contribution of noise (Figs. S11 and S12). The time window was positioned in the middle of the five-cycle ballistic wave. The square of the voltage was integrated across the time window. We calculated this value for each point in x-z-plane to construct an intensity map (Figs. E2 and E3).

The time when the acoustic burst arrives to the hydrophone changes as the hydrophone moves further away from the cylinder. We compensated for this by adding $t = vz$ to the examined time window. Here v is the speed of sound in the medium of study, and z is the distance from the cylinder along the z-axis. While moving along the x-axis, the varying distance was not compensated for since the shift in time of the ballistic wave was barely noticeable.

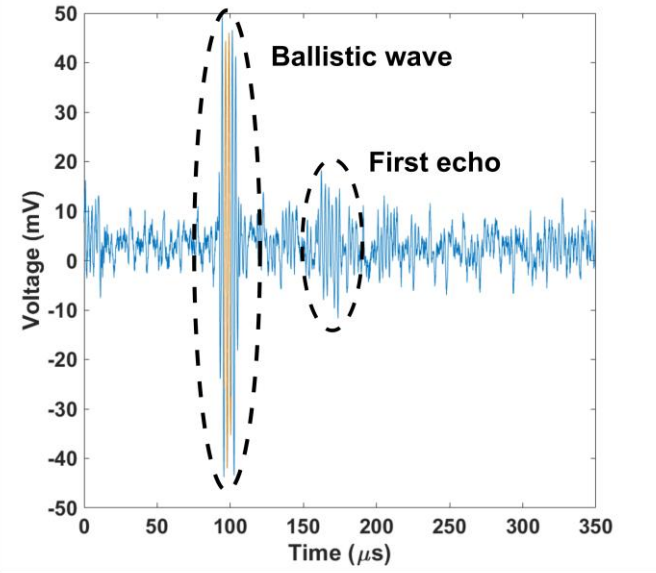

Supplementary Figure 11 - A-line in ethanol experiment at x = 0 mm and z = 2 mm. The ballistic wave and first echo arising from the ethanol-air boundary are indicated. A two-cycle time window (orange line) from the center of the ballistic wave was examined to minimize the contribution of noise (Fig. S12).

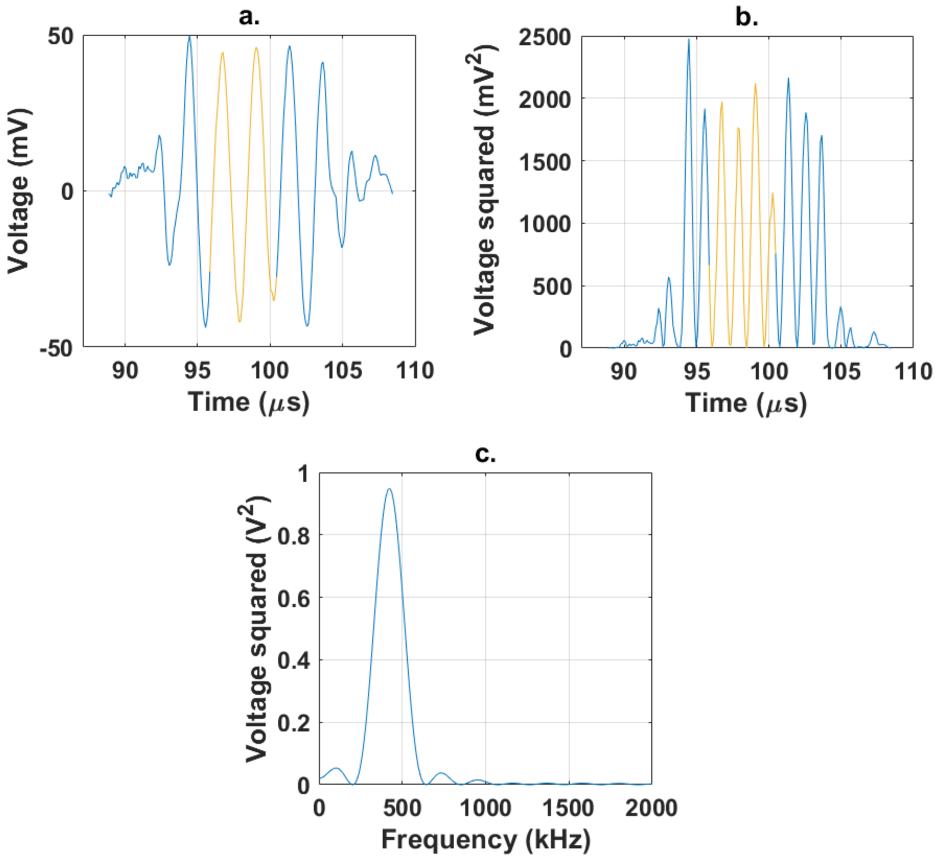

Supplementary Figure 12 - Analyzed data extracted from the A-line. a) Two-cycle time window picked from the center of the ballistic burst (Fig. S11). b) Square of the two-cycle window. A time integral of this curve represents a single pixel in the intensity plot figures (Figs. 1d, 2c, 3a, 3b, E2 and E3). c) Fourier transform of the acoustic signal (a). No significant contributions of higher harmonics are present.

UNCERTAINTY ANALYSIS

When we determined the phase velocity of the ultrasonic wave in different media, we used glass beakers. They marked the volume with 2 mm thick lines, so we estimated the distance uncertainty as $S(d) = 4\,mm$ (Fig. S7). The uncertainty in the speed of sound was calculated as slope uncertainty of the linear fit (Fig. S7).

Concerning the density, we used a precision scale to determine the weight of different volumes of liquid. We measured the liquid volume with a beaker featuring a volume uncertainty of 0.3 ml. The density uncertainty *S(ρ)* is:

$$S(\rho) = \frac{S(a)}{a^2}$$

where *a* is the slope in the linear fit of the experimental data (Fig. S7) and *S(a)* the standard deviation of the slope.

The ultrasonic phase velocity in a medium is:

$$v = \lambda f$$

where v is the phase velocity in the medium, λ the wavelength, and f the frequency of the ultrasonic excitation, respectively.

Since we accurately controlled the excitation frequency, we approximate the wavelength uncertainty as:

$$S(\lambda) = S(v)/f$$

where S(v) and S(λ) are the uncertainties in phase velocity and wavelength, respectively. When we compute the width of the jet (full width at half maximum divided by wavelength) we account for the uncertainty of the fit and the uncertainty of the wavelength.

$$y = FWHM/\lambda \quad S(y) = (1/\lambda)\sqrt{S^2(FWHM) + y^2 S^2(\lambda)}$$

where y is the width of the jet divided by the wavelength, $S^2(y)$ its variance, and $S^2(\lambda)$ the wavelength variance.

REFERENCES:

22. https://www.engineeringtoolbox.com/poissons-ratio-d_1224.html
23. https://www.makeitfrom.com/material-properties/Polystyrene-PS
24. Laradji, M. & Landau, D. P. Structural Properties of $Si_{1-x}Ge_x$ alloys: Monte Carlo simulation with the Stillinger-Weber potential. *Phys. Rev. B* **51**, 4894 (1995).
25. Plimpton, S. Fast Parallel Algorithms for Short-Range Molecular Dynamics. *J. Compu. Phys.* **117**, 1-19 (1995).
26. Stukowski, A. & Albe, K. Extracting dislocations and non-dislocation crystal defects from atomistic simulation data. *Modell. Simul. Mater. Sci. Eng.* **18**, 085001 (2010).

EXTENDED DATA

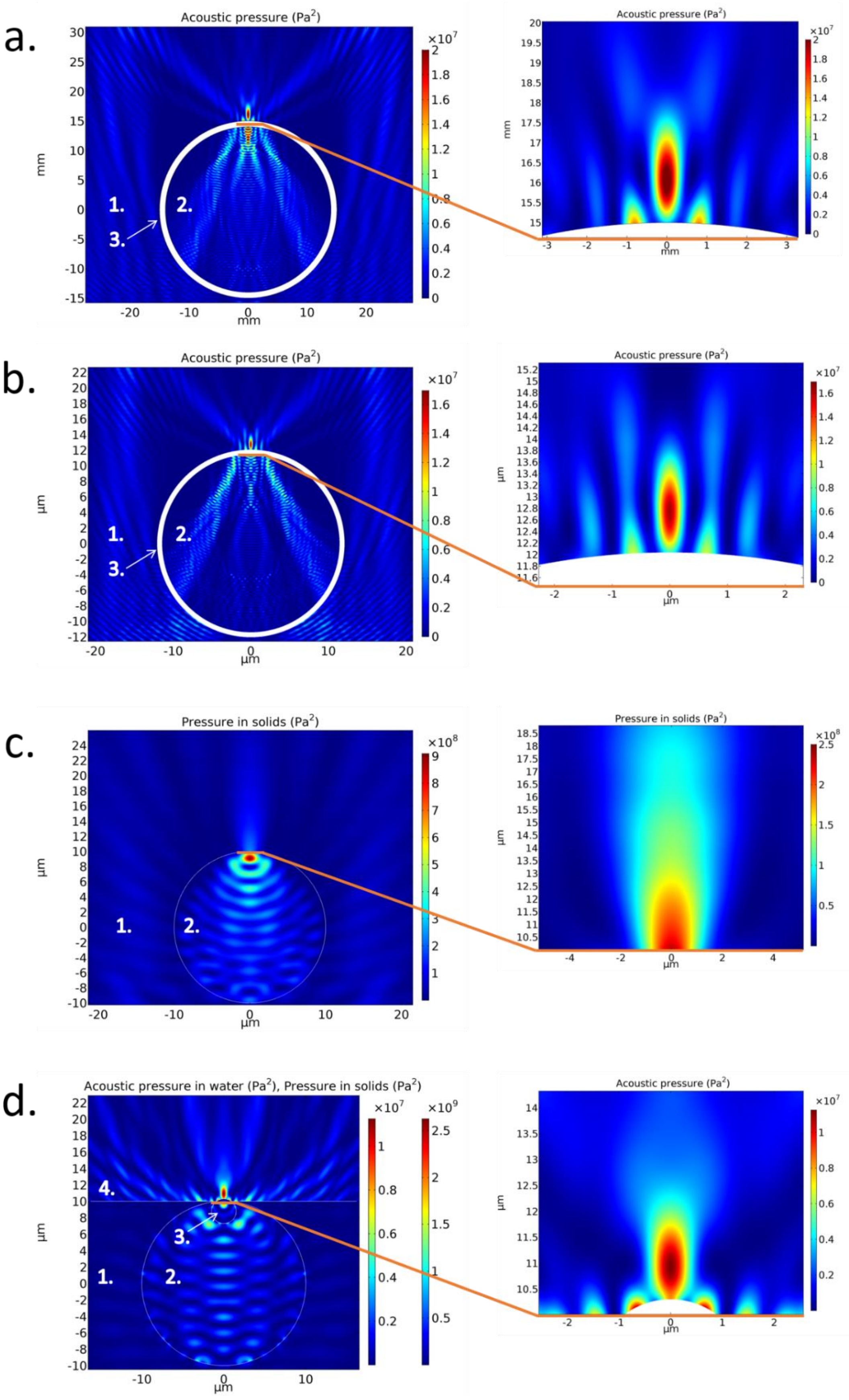

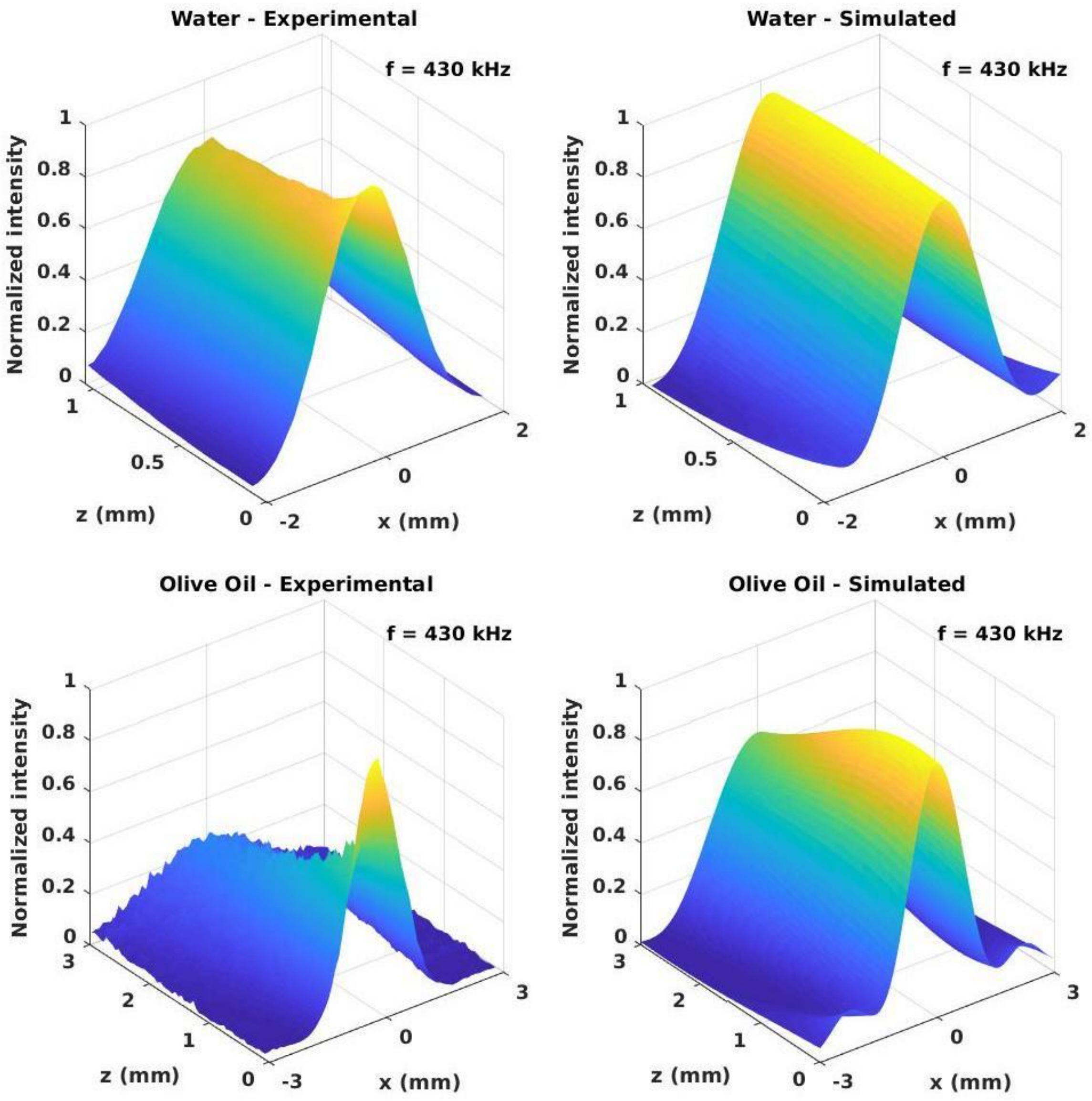
Water - Experimental
f = 430 kHz
Normalized intensity
z (mm)
x (mm)
Water - Simulated
f = 430 kHz
Normalized intensity
z (mm)
x (mm)
Olive Oil - Experimental
f = 430 kHz
Normalized intensity
z (mm)
x (mm)
Olive Oil - Simulated
f = 430 kHz
Normalized intensity
z (mm)
x (mm)

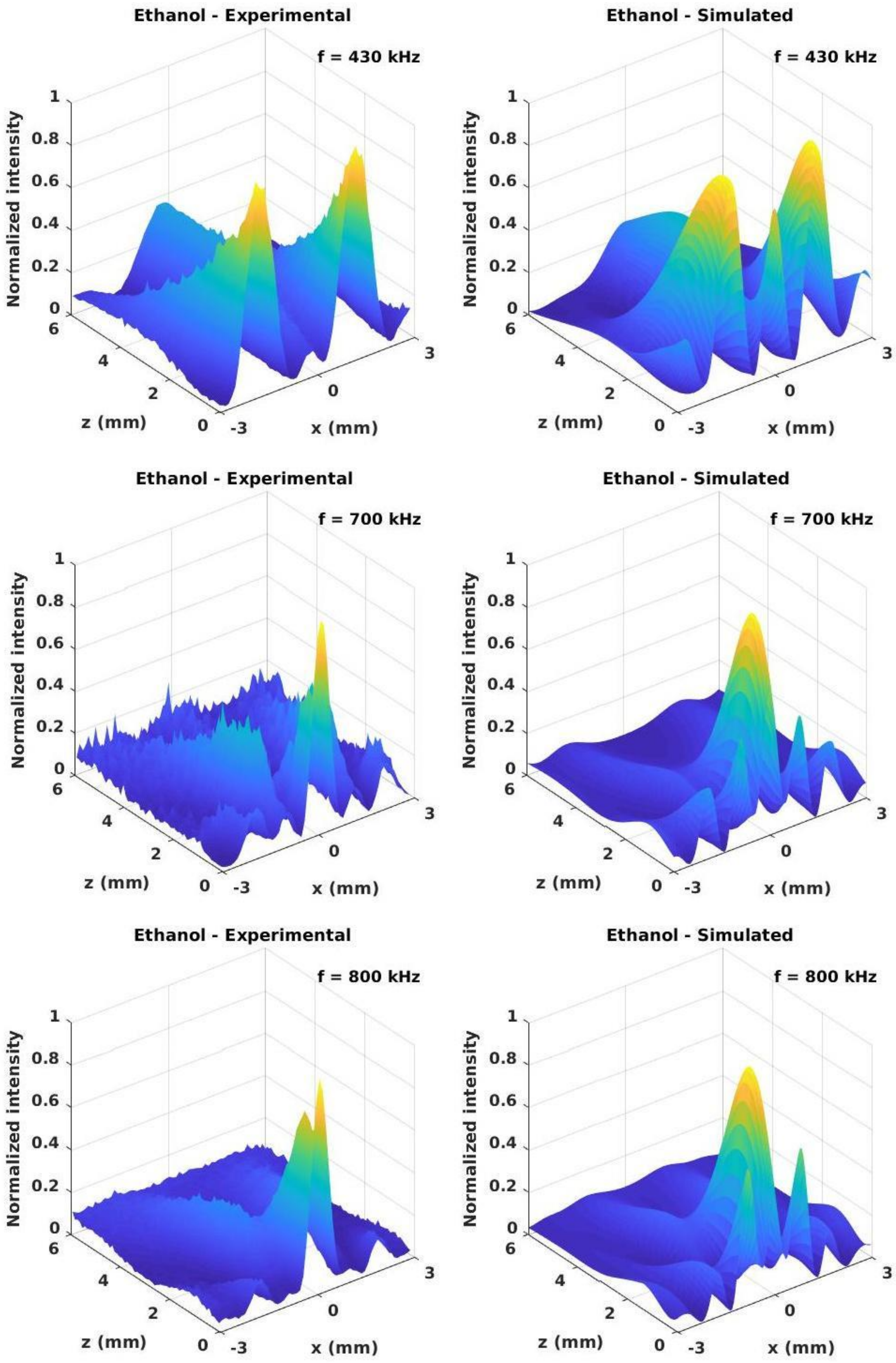

Ethanol - Experimental
f = 430 kHz
Ethanol - Simulated
f = 430 kHz
Ethanol - Experimental
f = 700 kHz
Ethanol - Simulated
f = 700 kHz
Ethanol - Experimental
f = 800 kHz
Ethanol - Simulated
f = 800 kHz
Normalized intensity
z (mm)
x (mm)
0
0.2
0.4
0.6
0.8
1
6
4
2
-3
3

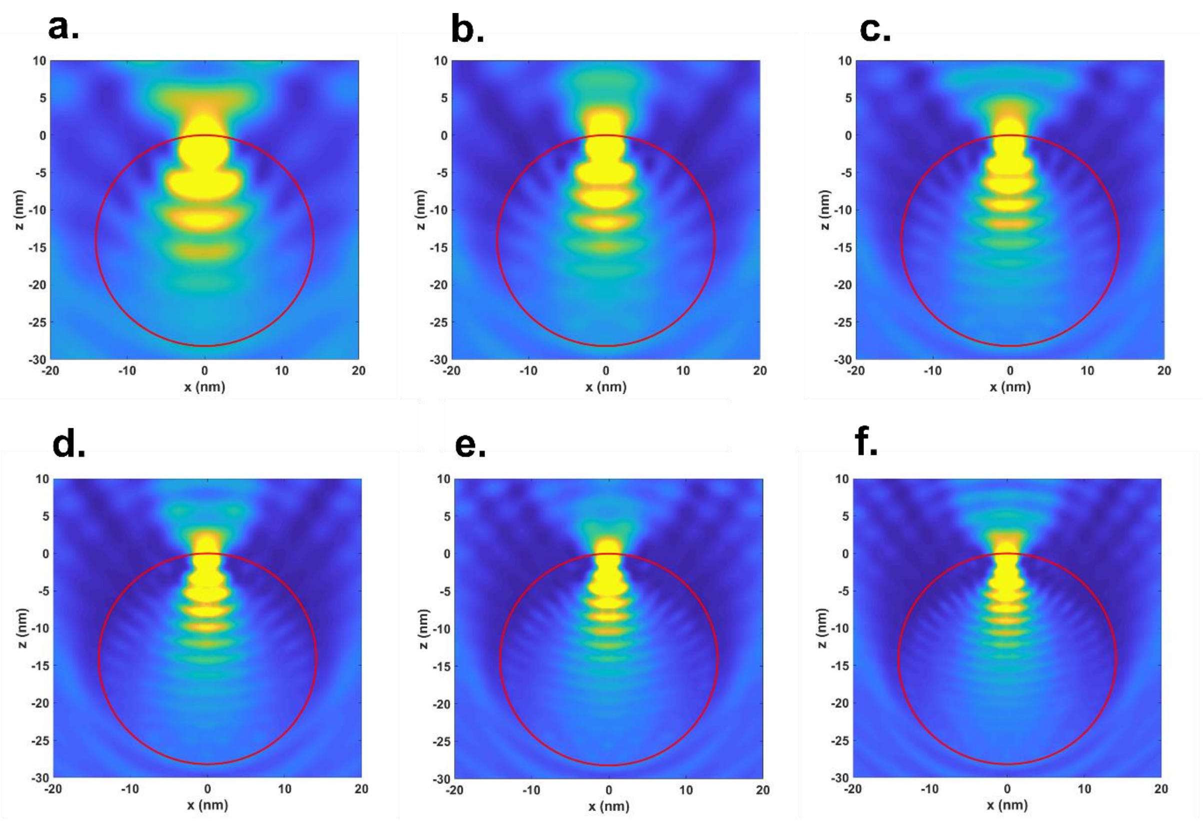
a.
b.
c.
d.
e.
f.
z (nm)
x (nm)

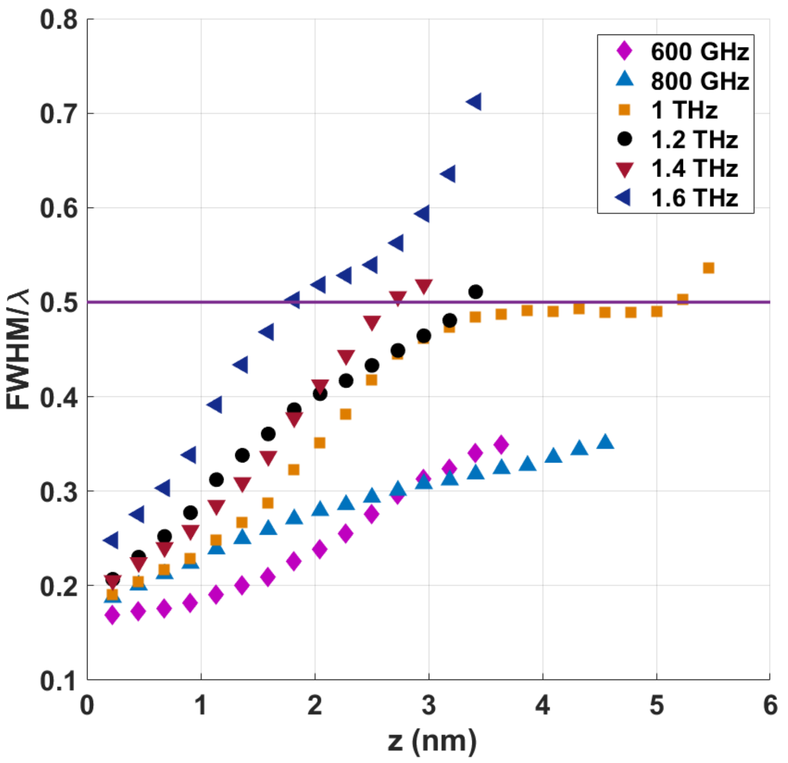
600 GHz
800 GHz
1 THz
1.2 THz
1.4 THz
1.6 THz
FWHM/λ
z (nm)

Extended Data 1 - Simulated intensity maps in a large window. Ethanol (1), perfluorinated oil (2) and polyethylene (3) at 800 kHz (a) and 1 GHz (b) respectively. (c) nickel (1) and gold (2) at 1 GHz. (d) nickel (1), gold (2), lead (3), and water (4) at 1 GHz. On the right side the color scale is adjusted to visualize the jet.

Extended Data 2 - Comparison between experimental and simulated intensity maps. In the left column, measured intensity maps of the acoustic jet in water and olive oil. In the right column, simulated intensity maps of water and olive oil experiments.

Extended Data 3 - Comparison between experimental and simulated intensity maps. In the left column, experimental intensity maps in ethanol with various frequencies. In the right column, corresponding simulated intensity maps.

Extended Data 4 – MD simulated intensity maps for various frequencies. (a) 600 GHz, (b) 800 GHz, (c) 1 THz, (d) 1.2 THz, (e) 1.4 THz, (f) 1.6 THz. The red circle marks the lens.

Extended Data 5 – Width of the MD simulated acoustic jet as a function of distance from the metamaterial lens for different frequencies. The purple line marks $\lambda/2$.
